# Vitamin D Toxicity Masquerading as Acute Pancreatitis

**DOI:** 10.7759/cureus.40189

**Published:** 2023-06-09

**Authors:** Aditya Kohli, Avantika Chawla, Saurabh Arora, Sanjay Kalra

**Affiliations:** 1 Internal Medicine, Dayanand Medical College and Hospital, Ludhiana, IND; 2 Endocrinology, Dayanand Medical College and Hospital, Ludhiana, IND; 3 Endocrinology, Bharti Hospital, Karnal, IND

**Keywords:** pancreatitis due to hypercalcemia, hypervitaminosis d, self medication, denosumab, 25-hydroxyvitamin d (25-ohd), acute pancreatitis, vitamin d toxicity

## Abstract

Patients and medical professionals are showing renewed interest in vitamin D supplementation as a result of increased knowledge of the positive health effects of vitamin D supplementation, the prevalence of vitamin D deficiency, and the easy availability of over-the-counter vitamin D pills. We present a case of acute pancreatitis following vitamin D toxicity due to the administration of doses exceeding the recommended dosage. A 61-year-old man presented to us with elevated pancreatic enzymes, increased 25-hydroxyvitamin D (25-OHD) levels, and deranged renal function tests. He was kept nil per oral and managed with intravenous fluids and denosumab injection. We advocate educating medical professionals about the frequently disregarded side effect of vitamin D supplementation. At the same time, it is critical to create awareness among the public about the harmful effects of self-medication.

## Introduction

Cholecalciferol (vitamin D3) is either consumed exogenously in the diet (from fish oil and fortified dairy products) or synthesized endogenously in the skin from 7-dehydrocholesterol by UV irradiation [1]. Vitamin D, a key regulator in calcium homeostasis and bone mineral metabolism, is an essential prohormone for maintaining equilibrium in the human body [2]. 25-OHD plays a vital role in parathormone (PTH) regulation, cell differentiation, and protection against a wide range of auto-immune conditions and cancer [3]. Rickets and osteomalacia, occurring due to a negative calcium balance, are the manifestations of vitamin D deficiency. However, in extremely rare conditions, overzealous correction of vitamin D can lead to hypervitaminosis D, subsequently causing vitamin D toxicity (VDT). Due to its wide therapeutic index, signs of vitamin D toxicity take time to appear and require an administration of mega-doses over an extended period [4]. Here, we report a unique case of acute pancreatitis following iatrogenic hypervitaminosis D as a consequence of the administration of 10 intramuscular injections of vitamin D (6,00,000 IU each) over six weeks.

## Case presentation

A 61-year-old male, a non-alcoholic and non-smoker with no comorbidities, presented to the emergency room with excruciating pain localized to the upper abdomen for three days, which was sudden in onset, radiating to the back, and worsened on lying down. It was associated with vomiting for three days. He denied any history of hematemesis, abdominal distension, constipation, chest pain, and dyspnea. The patient also had anorexia with limited dietary intake. On examination, his blood pressure was 120/70 mmHg, pulse was 102 beats per minute, and oxygen saturation of 98% on room air. On abdominal examination, mild tenderness was present in the epigastric region with no significant organomegaly. Chest, cardiovascular and neurological examination was essentially within normal limits.

Investigations

**Figure 1:**
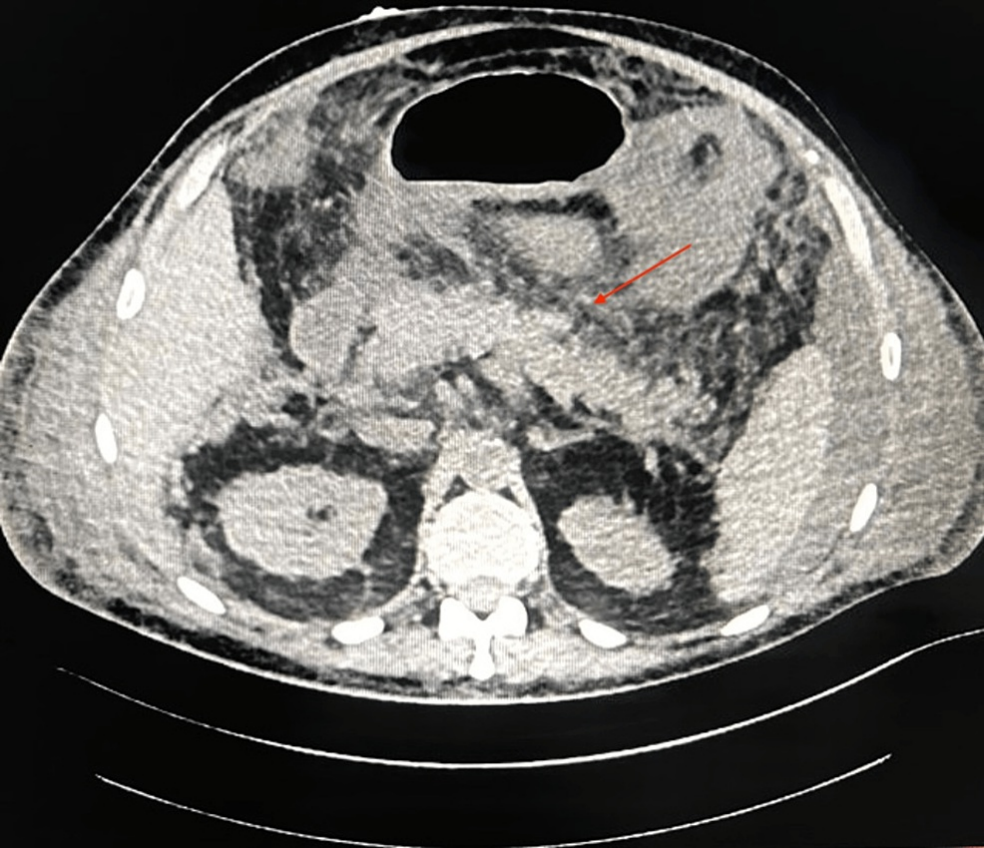
CT image showing diffuse enlargement of the pancreas with heterogenous enhancement and irregular contour of the pancreatic margins * Findings are marked with a red arrow

The patient had leukocytosis with persistent neutrophilia. He had significantly elevated amylase and lipase levels with deranged renal function tests. The additional diagnostic evaluation revealed elevated calcium levels with raised 25-hydroxyvitamin D and decreased PTH levels (Table 1). Immunoglobulin G4 (IgG4) levels were found to be normal, thus ruling out autoimmune pancreatitis. Serum protein electrophoresis did not reveal any sign of monoclonal gammopathy. Bone resorption markers were essentially within normal limits. Ultrasound abdomen revealed a distended gall bladder with an echo-free lumen and normal wall thickness. Common bile duct (CBD) and portal vein dimensions were normal. Intrahepatic biliary radicals (IHBRs) were not dilated. The pancreas was bulky in size, measuring approximately 20 mm in the head region with heterogeneous echotexture and decreased echogenicity. There was no evidence of peripancreatic collection. CT abdomen revealed diffuse enlargement of the pancreas with heterogeneous enhancement and irregular contour of the pancreatic margins suggestive of acute pancreatitis (Figure 1). In view of the altered sensorium, a non-contrast CT head was done to rule out any intracranial pathology, which was found to be normal.

**Table 1:** Blood evaluation (initial presentation, Day 4 of admission, and on discharge) 25-OHD: 25-hydroxyvitamin D; PTH: Parathormone; CRP (Q): C-reactive protein (quantitative); ACE: Angiotensin-converting enzyme; HDL-C: High-density lipoprotein cholesterol; LDL-C: Low-density lipoprotein cholesterol; VLDL-C: Very low-density lipoprotein cholesterol; N.A: Not available

Labs	On Admission	Day 4 post denosumab therapy	On discharge	Reference range
Total leucocyte count	17,100/mm^3^	14,500/mm^3^	11,300/mm^3 ^	4000-11,000/mm^3^
Hemoglobin	12.1 gm/dl	11.8 gm/dl	12.0 gm/dl	12-16 gm/dl
Platelet count	2,30,000/mm^3^	2,32,000/mm^3^	2,35,000/mm^3^	1,50,000-4,50,000/mm^3^
RBC count	3,33,000/mm^3^	3,35,000/mm^3^	3,38,000/mm^3^	4,50,000-5,50,000/mm^3^
PCV	38%	35%	34%	45.0-55.0(%)
S. amylase	560 U/L	420 U/L	120 U/L	28-100 U/L
S. lipase	600 U/L	470 U/L	140 U/L	13-60 U/L
S. creatinine	5.1 mg/dl	3.6 mg/dl	1.6 mg/dl	0.7-1.2 mg/dl
S. urea	62 mg/dl	46 mg/dl	32 mg/dl	10.0-50.0 mg/dl
S. calcium	13.1 mg/dl	11.6 mg/dl	10.4 mg/dl	8.6-10.2 mg/dl
25-OHD	>174 ng/dl	>104 ng/dl	> 54 ng/dl	>30 ng/dl sufficient; 21-30 ng/dl insufficient; <20 ng/dl deficient
PTH	4.78 pg/ml	8.56 pg/ml	13.46 pg/ml	15.0-65.0 pg/ml
CRP (Q)	13.45 mg/L	10.42 mg/L	8.67 mg/L	0.0-6.0 mg/L
ACE	79.20 U/L	N.A	77.82 U/L	65.0-115.0 U/L
Total cholesterol	190 mg/dl	N.A	174 mg/dl	<200.00 mg/dl
Triglycerides	130 mg/dl	N.A	136 mg/dl	<150.00 mg/dl
LDL-C	98 mg/dl	N.A	87 mg/dl	<100.00 mg/dl
HDL-C	61 mg/dl	N.A	56 mg/dl	>50.00 mg/dl
VLDL-C	26 mg/dl	N.A	28 mg/dl	<30.00 mg/dl

Differential diagnosis

Blood investigations ruled out primary hyperparathyroidism and multiple myeloma. Sarcoidosis was kept as a possible differential due to hypercalcemia. The absence of mediastinal lymphadenopathy, normal lung parenchyma, and normal angiotensin-converting enzyme (ACE) levels argued against the diagnosis of sarcoidosis. Since the patient's lipid profile was within normal range, hypertriglyceridemia was ruled out as the cause of pancreatitis. A detailed medication history was obtained and none of the drugs were identified as potential triggers for acute pancreatitis. 25-OHD levels turned out to be significantly elevated, indicating vitamin D toxicity as a cause of hypercalcemia. Elevated serum amylase and lipase levels were suggestive of acute pancreatitis. A final diagnosis of acute pancreatitis with hypercalcemia was made, secondary to vitamin D toxicity.

Treatment and outcome

On initial admission, complete bowel rest was maintained by keeping the patient nil per oral, and the patient was managed conservatively with intravenous fluids. Steroids were not given in view of pancreatitis, and the patient was not responsive to calcitonin. Due to their renal elimination, bisphosphonates could not be administered in view of deranged renal function. Subsequently, 60 mg of denosumab was given subcutaneously, and the patient’s condition improved. Serum calcium levels showed a decreasing trend within four days of denosumab administration and almost touched baseline at the time of discharge. The patient was then discharged in a stable condition. After three weeks, the patient was asymptomatic with normal serum amylase and lipase levels and serum calcium level was within normal limits.

## Discussion

Vitamin D is a prohormone that is metabolized in the liver to 25-hydroxyvitamin D and then in the kidney to its biologically active metabolite 1,25-dihydroxy vitamin D [5]. It is responsible for maintaining mineral homeostasis in the body by optimizing calcium and phosphorus absorption and for adequate bone formation [6]. Osteoclast proliferation via the nuclear factor kappa B ligand (RANKL) pathway, protection against carcinoma, and a variety of autoimmune diseases constitute the non-calcium-related functions of vitamin D in our body [1-3]. Deficiency of vitamin D (25-OHD), a widely prevalent condition, can lead to hypocalcemia and subsequently, bone-related disorders, which are treated with oral vitamin D supplementation. The recommended pharmacological dose is oral administration of 50,000 IU per week for six to eight weeks [2]. The guidelines of the Food and Nutrition Board of the USA provide a recommendation of 2000 IU as the highest vitamin D intake for consumption in healthy adults without risk of hypercalcemia [7]. The exponential rise in vitamin D measurement and intake due to an increase in laboratory testing has led to its indiscriminate use, potentially leading to increased incidence of its toxicity and associated sequelae of complications such as acute kidney injury and acute pancreatitis [8]. Although a rare entity, vitamin D toxicity (VDT) can be life-threatening with significant morbidity [9]. Self-medication and injudicious use of vitamins have been on the rise due to widespread availability. Although there are certain age groups requiring vitamin D supplementation, e.g. exclusively breastfed children and the elderly population who are at risk for hypovitaminosis, it is beneficial only until the target serum levels are maintained [10]. Clinical manifestations of VDT include hypercalcemia, hypercalciuria, kidney stones, polyuria, polydipsia, ectopic calcification of soft tissues, nausea, vomiting, anorexia, constipation, headache, and acute pancreatitis [11]. The association between acute pancreatitis and vitamin D toxicity has rarely been described. Our patient had a classical clinical presentation of acute pancreatitis, further supported by increased amylase and lipase levels following vitamin D toxicity. Denosumab was given to lower the patient’s calcium levels, as both steroids and bisphosphonates were contraindicated. Unlike bisphosphonates, the pharmacokinetics of denosumab are unaffected by renal function impairment because it is eliminated in the reticuloendothelial system. Denosumab is not nephrotoxic as opposed to bisphosphonates, making it a more viable therapeutic choice in cases of acute kidney injury [12]. Denosumab prevents osteoclast activation by competitively blocking the RANKL pathway, which has a direct impact on the pathophysiological mechanism of vitamin D overdose [13]. This case report, therefore, highlights the role of denosumab in the management of hypercalcemia due to vitamin D toxicity in a patient with concomitant acute pancreatitis and acute kidney injury.

## Conclusions

Despite its wide therapeutic index, consumption of large amounts of vitamin D can result in the development of vitamin D toxicity. The need of the hour is to increase awareness among the general public and healthcare providers about the correct usage of vitamin D. It is crucial to follow the recommended dosage guidelines and consult with a healthcare professional before starting any new supplement regimen, especially if there are any underlying health conditions. The patients should be counseled about the risk spectrum accompanying self-medication, inadvertent use of over-the-counter vitamins, and their potentially deleterious effects.
